# Cargo-Dependent Targeted Cellular Uptake Using Quaternized Starch as a Carrier

**DOI:** 10.3390/nano13131988

**Published:** 2023-06-30

**Authors:** Yossi Blitsman, Chen Benafsha, Nir Yarza, Jonathan Zorea, Riki Goldbart, Tamar Traitel, Moshe Elkabets, Joseph Kost

**Affiliations:** 1 Department of Chemical Engineering, Ben-Gurion University of the Negev, Beer-Sheva 84105, Israel; blitsman@post.bgu.ac.il (Y.B.); chenbere@post.bgu.ac.il (C.B.); goldbart@post.bgu.ac.il (R.G.); tamluz@post.bgu.ac.il (T.T.); 2 The Shraga Segal Department of Microbiology, Immunology and Genetics, Faculty of Health Sciences, Ben-Gurion University of the Negev, Beer-Sheva 8410501, Israel; j.zorea@gmail.com (J.Z.); mosheelkabets@gmail.com (M.E.)

**Keywords:** targeted drug delivery, quaternized starch, cellular uptake, self-assembly complexes, plasmid DNA, small interfering RNA, phosphatidylinositol (3,4,5)-trisphosphate

## Abstract

The tailored design of drug delivery systems for specific therapeutic agents is a prevailing approach in the field. In this paper, we present a study that highlights the potential of our modified starch, Q-starch, as a universal and adaptable drug delivery carrier for diverse therapeutic agents. We investigate the ability of Q-starch/cargo complexes to target different organelles within the cellular landscape, based on the specific activation sites of therapeutic agents. Plasmid DNA (pDNA), small interfering RNA (siRNA), and phosphatidylinositol (3,4,5)-trisphosphate (PIP3) were chosen as representative therapeutic molecules, acting in the nucleus, cytoplasm, and membrane, respectively. By carrying out comprehensive characterizations, employing dynamic light scattering (DLS), determining the zeta potential, and using cryo-transmitting electron microscopy (cryo-TEM), we reveal the formation of nano-sized, positively charged, and spherical Q-starch complexes. Our results demonstrate that these complexes exhibit efficient cellular uptake, targeting their intended organelles while preserving their physical integrity and functionality. Notably, the intracellular path of the Q-starch/cargo complex is guided by the cargo itself, aligning with its unique biological activity site. This study elucidates the versatility and potency of Q-starch as a versatile drug delivery carrier, paving the way for novel applications offering targeted delivery strategies for potential therapeutic molecules.

## 1. Introduction

Various bioactive molecules, including small-molecule drugs, antibodies, proteins, RNAs, and DNAs, have been widely studied for the last few decades [1,2,3,4,5]. In order to achieve the desired therapeutic effect, the bioactive molecules need to overcome several hurdles on their way to the specific intracellular target site. Low stability is the first hurdle faced by many bioactive agents [6,7,8]. Next, when approaching the target cell, the bioactive agents encounter the cell’s membrane, which acts as a selective barrier to the size and charge of specific molecules [9,10,11,12]. If the molecule is able to pass through the cell membrane, it then encounters further obstacles, including endosomal escape, degradation by cell enzymes, and the nuclear membrane [13,14,15]. Overcoming these obstacles is possible using drug delivery systems that stabilize the bioactive agents and change their surface chemistry, charge, topography, and morphology. In addition, drug delivery systems enable the release of drugs as a response to biological stimuli, including pH, temperature, reduction–oxidation, and enzymes [16,17,18,19,20,21,22,23,24]. 

Polysaccharides are long-chain polymeric carbohydrates found in animals, plants, and microbial sources; they serve as an energy source and storage in addition to their structural role [25,26]. Various polysaccharides have been used for centuries in foodstuffs; in recent decades, they have gained new attention in the field of drug delivery systems [27,28,29,30]. The use of polysaccharides in biological applications is advantageous because of their low toxicity, safety, biodegradability, biocompatibility, and high stability [31,32,33,34,35,36]. In addition, polysaccharides are inexpensive and exist in various structures that can be easily modified chemically and biochemically, allowing the binding of desired molecules [37,38,39,40,41]. Moreover, studies have shown that polysaccharides exhibit various beneficial biological attributes, including immunoregulatory, anti-inflammatory, hypoglycemic, antibacterial, antioxidant, and antitumor effects [31,42,43]. Chitosan, hyaluronic acid, dextran, starch, and cellulose are a partial list of polysaccharides used as drug delivery systems for various therapeutic molecules such as doxorubicin, furosemide, therapeutic peptides and proteins, RNAs, and DNAs [44,45,46,47,48]. Adjusting the polysaccharide carrier according to the drug’s properties is frequently necessary to create a polysaccharide drug delivery system that enables high drug loading, stability, and targeting ability. It is usually achieved by chemical modification of its backbone using reagents such as amines, disulfide bridges, or crosslinking by ionic bonding [41,49,50]. 

Modified alginate, for example, has been used to prepare microcapsules through self-crosslinking by a template method targeting the nucleus [51]. Another example of using polysaccharides as a drug system to target the cytoplasm involves the use of polyelectrolyte complexes composed of two polymers with opposite charges, e.g., cationic polysaccharides, such as chitosan, together with anionic polysaccharides such as hyaluronic acid. These polyelectrolyte complexes serve as a drug delivery system for small interfering RNA (siRNA) active in the cellular cytoplasm [45,52]. Each polysaccharide-based drug delivery system has its advantages, but to deliver the bioactive agent to the target site, tailoring the polysaccharide as a specific drug delivery system must be carried out. 

In light of the above, we wondered if a more general approach might be feasible. Could the same polysaccharide carrier serve different biological agents for targeting different intracellular organelles? In this study, starch was modified using a quaternary amine group to form a positively charged carrier [53,54,55]. The quaternized starch (Q-starch) carrier can self-assemble with negatively charged therapeutic molecules to form a nano-delivery system (complex) that can overcome charge- and size-related cellular barriers [56,57]. In this study, we investigated three different negatively charged therapeutic molecules (cargo): plasmid DNA (pDNA); small interfering RNA (siRNA); and phosphatidylinositol (3,4,5)-trisphosphate (PIP3). Because each of these therapeutic molecules needs to target different sites in the cell (nucleus, cytoplasm, and cell membrane, respectively) to achieve their therapeutic effect, we studied these complexes’ intracellular paths for different types of cell lines to assess whether each of the complexes would reach different organelles in the target cell, depending on the cargo itself.

The three complex types were characterized, and their cellular uptake was observed in human immortalized keratinocytes (HaCAT), basal cell carcinoma (BCC), and melanoma cell lines. Next, after verifying that the complexes were able to reach their target sites, their biological activity was examined in different relevant cell lines. The Q-starch/pDNA complex was analyzed by transfection of the green fluorescent protein (GFP) gene to the nucleus of HaCAT cells using confocal microscopy. Q-starch/siRNA complexes were examined using siRNA that targets the GLI2 gene in basal cell carcinoma (BCC) cells, overexpressing the GLI2 gene [58,59]. GLI2 gene silencing efficiency was evaluated by Real Time PCR analysis of GLI2 gene mRNA expression. Q-starch/PIP3 complex activity (activation of AKT protein) was investigated on the HaCAT cell line. Cells were first treated with PI3K inhibitor BYL719 followed by the Q-starch/PIP3 complex [60,61]. AKT activation was then evaluated by Western blot.

## 2. Materials and Methods

### 2.1. Starch Quaternization

Quaternary starch synthesis was carried out as previously described by Amar Lewis et al. [59]. Briefly, soluble hydrolyzed potato starch (Mw 26,765 Da) was dissolved in sodium hydroxide solution (0.19 g/mL) to obtain a 50 mg/mL starch concentration. The solution was then stirred (100 rpm, AGE magnetic stirrer, Velp Scientifica, Usmate Velate, Italy) for 40 min at room temperature. The quaternization reagent, 3-chloro-2-hydroxypropyltrimethyl-ammonium chloride (CHPTAC) solution, was mixed with double-distilled water (DDW, 18.3 MΩ·cm) to a final concentration of 0.32 g/mL and was then added slowly to the starch solution. The reaction volume was continuously stirred for 24 h at room temperature. An acidified (1% vol. HCl) mixture of ethanol and acetone (1:3 % vol.) was added to the starch solution in a ratio of 4:1 to precipitate the reaction product. The residue was washed four times with 80% vol. ethanol, dissolved in a small volume of DDW, and poured into a 14 KDa molecular weight cut-off dialysis tube. The dialysis tube was placed in a vessel containing 5 L of DDW for 48 h of dialysis, during which time the water was replaced with fresh water four times. Finally, the dialyzed product was freeze-dried and lyophilized for 72 h to obtain the purified quaternized (Q-starch) product.

### 2.2. Quaternized Starch Labeling

Q-starch molecules were labeled using 5-(4,6-Dichlorotriazinyl) amino fluorescein (5-DTAF) to be visualized by fluorescent microscopy. A 100 mg amount of Q-starch was dissolved in 3 mL DDW. The pH was adjusted to 11–12 with 1 M NaOH, and the mixture was stirred (100 rpm, AGE magnetic stirrer, Velp Scientifica) for 30 min at room temperature. Dissolved 5-DTAF (7.5 mg of 5-DTAF in 0.3 mL DMSO) was added to the Q-starch solution. The solution was stirred (100 rpm, AGE magnetic stirrer, Velp Scientifica) at room temperature under light exclusion for 24 h. The solution was neutralized with 0.1 M HCl and poured into a 14 KDa molecular weight cut-off dialysis tube. The dialysis tube was placed in a vessel containing PBS solution (pH 7.4) for 72 h and then for 48 h in DDW (18.3 MΩ∙cm). To obtain purified labeled Q-starch^DTAF^, the dialyzed product was freeze-dried and lyophilized for 72 h so that complete dryness was obtained.

### 2.3. Quaternized Starch Chemical Analysis

Starch quaternization was confirmed by Fourier transform infrared spectroscopy (FTIR), ^1^H NMR, and elemental analysis (EA). FTIR was obtained in a Thermo-Nicolet FTIR spectrophotometer (Model-Nicolet iS50 FTIR), and samples were prepared in the form of potassium bromide (KBr) pellets. NMR spectra were recorded on a 500 MHz Brucker spectrometer using D_2_O as a solvent. Nitrogen content (N% weight) in the Q-starch product was measured by the elemental analysis method using a Thermo Scientific FLASH 2000 NC Analyzer.

### 2.4. Q-Starch/Cargo Complex Preparation

Plasmid pEGFP-C2 encoding for green fluorescence protein (GFP) was amplified in our laboratory in *Escherichia coli DHα* and purified using a HiSpeed Plasmid Maxi Kit from QIAGEN. Noncoding siRNA NC5 (siNC5) and siRNA^GLI2^ were purchased from Integrated DNA Technologies Inc. (IDT, ref nos. 144338509 and 274197214, respectively). The siNC5 sense sequence was 5′-CAUAUUGCGCGUAUAGUCGCGUUAG-3′, whereas the siRNAGLI2 sense sequence was 5′CAGAAUGAGGCUCGUAAUGGUACCUUC-3′. PIP3 (P-3916) was purchased from Echelon Bioscience.

Complexes of different cargos were prepared at N/P molar ratios of 1 to 3; N/P is the molar ratio between the positively charged amine groups on the quaternized starch (N) and the negatively charged groups (e.g., phosphate groups) on the cargo (P). N/P ratios between 1 and 3 were characterized in order to determine the ratio at which the complex’s zeta potential and size were most suitable for entering the cell. All the cargo molecules, i.e., pDNA, siRNA, and PIP3, were dissolved in Dnase- and RNase-free water to concentrations of 330 μg/mL, 20 μM, and 0.6 μg/μL, respectively. Q-starch was dissolved in Dnase- and RNase-free water to a concentration of 0.4 mg/mL and added in aliquots to the different cargo solutions. For example purposes, the procedure for preparing Q-starch/cargo complexes at N/P 2 is presented as follows: For Q-starch/pDNA complexes, 27.2 μL of Q-starch 0.4 mg/mL was introduced into tubes containing 11.5 μL of 330 μg/mL pDNA solution. For Q-starch/siRNA complexes, 6.6 μL of Q-starch 0.4 mg/mL was introduced into tubes containing 1.0 μL of 20 μM siRNA solution. For Q-starch/PIP3 complexes, 4.2 μL of Q-starch 0.4 mg/mL was introduced into tubes containing 1.0 μL (0.6 mg/mL) of 60-seconds-sonicated (Cole-Parmer Ultrasonic Cleaner) PIP3 solution. The mixed solution was gently vortexed (Vortex-Genie 2, Scientific Industries, Inc., Bohemia, NY, USA) and incubated at room temperature for 40–60 min to allow the complex to form by self-assembly. For different N/P ratios, the volume of starch solution was changed accordingly. 

### 2.5. Q-Starch/Cargo Complex Characterization

The hydrodynamic radius of the complexes was measured by DLS. Q-starch/cargo complexes were prepared as described above, reaching concentrations of 10 μg/mL plasmid-DNA, 250 nM siRNA, and 250 nM PIP3 in Eppendorf tubes containing the complexes at a final volume of 1 mL. The spectra were collected using a CGS-3 (ALV, Langen, Germany) goniometer. The laser was powered with 20 mW at the He-Ne laser line (632.8 nm). Correlograms were calculated by an ALV/LSE 5003 cross-correlator, oriented at 90°, for 10 s, 30 times, at 25 °C. Each sample was placed into a thin-walled cylindrical glass cuvette and then placed in a vat filled with toluene as the optical matching fluid. A sample of each complex was then measured. 

The zeta potential of the complexes, indicative of each complex’s surface charge, was determined by a zeta sizer. The Q-starch/cargo complexes were prepared as mentioned, reaching a concentration of 5 μg/mL plasmid-DNA, 50 nM siRNA, and 50 nM PIP3 in an Eppendorf tube at a final volume of 1 mL. Samples were transferred to a U-tube cuvette (DTS1070C, Malvern, UK) for measurement of zeta potential using Zetasizer (ZN-NanoSizer, Malvern, UK). Each free cargo sample, free Q-starch, and samples of complexes at different N/P ratios in a range of 1–3 were measured in an automatic mode at 25 °C. The Smoluchowski model was used to calculate the zeta potential. 

The morphology of the complexes, as well as confirmation of their size, was evaluated by cryo-TEM. The Q-starch/cargo complexes were prepared to reach final concentrations of 10 μg/mL of plasmid-DNA, 250 nM siRNA, and 250 nM PIP3 in Eppendorf tubes at a final volume of 1 mL. Specimens of Q-starch/cargo complexes were prepared on a copper grid coated with a perforated lacey carbon 300 mesh (Ted Pella Inc., Redding, CA, USA) under controlled temperature (cryogenic temperature). A 3 µL drop of the analyzed solution was applied to the grid and blotted with filter paper to form a thin liquid layer. The blotted samples were immediately and automatically plunged into liquid ethane at its freezing point (−183 °C) using Plunger (Leica EM GP). The specimens were transferred into liquid nitrogen for storage. Samples were analyzed using FEI Tecnai 12 G2 TEM at 120 kV with a Gatan cryo-holder maintained at −180 °C. Images were recorded on a slow-scan cooled charge-coupled device camera (Gatan, Pleasanton, CA, USA) at low-dose conditions to minimize electron-beam radiation damage. The recording was carried out using the Digital Micrograph software package.

### 2.6. Cell Culture Handling

The human immortalized keratinocytes (HaCAT) cell line, basal-cell carcinoma (BCC) cell line, and melanoma cell line (A375) were selected as in vitro models in order to study the complexes’ target sites and their biological activity in relevant cell lines. HaCAT cells were grown in MEM Eagle growth medium containing 4.5 mM glucose, 10% vol. fetal bovine serum (FBS), 1% vol. L-glutamine (2 mM), and 1% vol. penicillin-streptomycin (100 µg/mL penicillin and 100 µg/mL streptomycin). BCC cells were cultured in DMEM growth media containing 10% vol. FBS and 1% vol. penicillin-streptomycin. Melanoma cells were cultured in RPMI growth media containing 1% vol. penicillin-streptomycin, 1% vol. L-glutamine, 1% vol. sodium pyruvate solution, 0.025% vol. HEPES buffer 1M, 0.125% vol. Nystatin suspension, and 10% vol. FBS. All cells were grown in a 75 cm^2^ flask. 

Splitting of cells was performed with 2–3 mL of trypsin-EDTA every 3–4 days into 3–4 flasks to prevent high cell confluence and overpopulation of the culture. Following splitting, the trypsin was neutralized with 10 mL of growth medium. Cells were then well pipetted to ensure a homogeneous suspension and counted by the Countess™ II FL Automated Cell Counter. Trypan blue stain was used for counting viable cells by mixing 20 μL of the cell’s suspension extract with 20 μL of 0.4% trypan blue solution (1:1 *v*/*v* ratio). All cell-culture procedures were performed inside a laminar-flow hood that was sterilized before each procedure by UV light and by wiping the working area with 70% ethanol.

### 2.7. Determination of Intracellular Paths of Complexes 

For the cellular uptake, live confocal microscope samples of 5∙10^4^ HaCat cells/well were seeded in an eight-well plate (Ibidi µ-Slide 8-Well Glass Bottom 80827) for 24 h prior to the beginning of the experiment. On the day of treatment, cells were washed twice with PBS and stained with one or more of the following dyes according to the manufacturer’s instructions: NucBlue (nucleus dye, Ex. 360 nm, Invitrogen, Waltham, MA, USA); ViaFluor488 (cytoplasm dye, Ex. 488 nm, Biotium); or CellBrite™ (membrane dye, Ex.633 nm, Biotium). After staining, cells were washed twice with PBS, and 300 µL from each of the complexes was added to the cells to final concentrations of 4 mg/mL of pDNA, 100 nM of labeled noncoding siRNA (siRNA NC5Cy5), and 500 nM of PIP3. Cells were incubated at 37 °C and 5% CO_2_ for a total of 4 h. Images were taken after 4 h using ZEN software on the LSM-880 confocal system (ZEISS, Oberkochen, Germany). A Plan-Apochromat 40×/1.4 oil DIC M27 and 10× objective were used, and excitation was carried out with solid-state and argon lasers, as well as T-PMT for transmission light detection by a 32-channel GaAsP array. Images were acquired with EF6 and EF7 filters. The intracellular paths of Q-starch/siRNACy5 complexes were determined in melanoma A375 and BCC cell lines, in addition to HaCAT cells.

In order to track the complexes’ intracellular paths by microscopic means, it was necessary to label the cargo or the carrier with fluorescent dyes. Detection of Q-starch/siNC5^Cy5^ complexes was carried out with a custom-designed labeled siNC5 (sense -5′Cy5-CUAACGCGACUAUACGCGCAAUAUGGU-3′, IDT, excitation at 633 nm). For Q-starch/pDNA and Q-starch/PIP3 complexes, detection of fluorescently labeled Q-starch^DTAF^ was carried out (excitation at 488 nm). Q-starch/cargo complexes were prepared at an N/P ratio of 5 for Q-starch/pDNA and an N/P ratio of 2 for both Q-starch/siRNA^Cy5^ and Q-starch^DTAF^/PIP3. These ratios were found to be the optimal N/P ratios to achieve high biological activity for these cell lines, based on previous work performed in our lab [49,50]. Labeling of the Q-starch/cargo complexes is described in Table 1.

### 2.8. Biological Activity Evaluation 

#### 2.8.1. pDNA: pGFP Transfection 

The Q-starch/pGFP transfection was carried out as previously described by Sieradzki et al. [55]. Twenty-four hours before transfection, 5∙10^4^ HaCAT cells/well were seeded in Ibidi µ-Slide 8-Well Glass Bottom culture plates and grown to attain ~80% confluence on transfection day. Complexes of Q-starch/pGFP at a final concentration of 4 mg/mL pDNA, according to the N/P ratio of 5, were prepared in a serum-free media. A total of 300 µL of complex solution was added to the culture plate and incubated for 4 h at 37 °C under a 5% CO_2_ atmosphere. After 4 h, the media were replaced with fresh media containing serum; immediately after this, live images of the cells were taken using live confocal microscopy (LSM-880) for the detection of GFP expression. Untreated cells were used as a negative control.

#### 2.8.2. siRNA: GLI2 Gene Silencing 

BCC cells were seeded in a 6-well plate for 24 h in DMEM growth media at a concentration of ~1.8 × 10^5^ cells/well to reach a confluence of approximately 60–70%. On the day of transfection, Q-starch/siRNA complexes, either with non-targeting siRNA (siRNA NC5) or with siRNA targeting GLI2 transcription factor (siRNA^GLI2^), were prepared at an N/P ratio of 2 (based on previous work [62]). Cell media were aspirated, cells were washed twice with PBS, and complexes were placed on top of the cells at siRNA concentrations of 35 nM at a final volume of 2 mL/well. Amounts of 2 mL/well of serum and antibiotic-free media were placed for 4 h to allow the complexes’ cellular uptake (based on our results). Four hours after transfection, the media were changed to contain FBS and antibiotics. Thereafter, cells were incubated with fresh media at 37 °C and 5% CO_2_ for 72 h. Untreated cells were used as a negative control. 

Following incubation, a series of RNA extraction and purification steps were performed using the PureLinkTM RNA Mini Kit (12183020, Invitrogen) to extract the cells’ RNA. Samples were then prepared using the QuibitTM RNA BR Assay Kit (Q10210, Invitrogen), and RNA concentration was measured by the Qubit 3.0 Fluorometer (Q33216, Life Technologies). In line with the manufacturer’s instructions, a master mix solution was prepared on ice by gently mixing nuclease-free water, buffer, and enzymes from a qScript^TM^ cDNA Synthesis Kit (95047-100, Quanta-bio, Beverly, MA, USA) in a vol. % ratio of 5:4:1 (i.e., 50 µL of nuclease-free water was mixed with 40 µL buffer and 10 µL enzymes). RNA samples were prepared for PCR analysis as follows: 10 µL from the master mix was added to 20 µL of the RNA samples in a PCR tube to reach a concentration of 20 ng/µL RNA, then it was placed in a PCR (Peltier-based Thermal Cycler MGL96+, MyGeneTM L Series, HY-Labs, Rehovot, Israel) to obtain cDNA. 

The produced cDNAs were subjected to real-time polymerase chain reaction (RT-PCR) testing (StepOnePlus™ Real-Time PCR System, 4376600, Rhenium) to determine gene silencing by quantifying GLI2’s mRNA expression. cDNAs were diluted from the produced PCR stock to 10 ng/µL (1:2 with nuclease-free water). A stock solution was prepared on ice by gently mixing nuclease-free water, master mix solution (TaqMan™ Fast Advanced Master Mix, 4444557, Applied Biosystems, Waltham, MA, USA), and TaqMan probe and primers (TaqMan^®^ Gene Expression Assay, INV/SM: GLI2:Hs01119974, AB-4331182, HPRT:Hs99999909, Rhenium) in a ratio of 6:10:1, i.e., 60 µL of nuclease-free water was mixed with 100 µL from the master mix and 10 µL of probe and primers assay. Then, 3 µL of cDNA sample was added to a well in a MicroAmp™ Fast Optical 96-Well Reaction Plate with Barcode (0.1 mL, 4346906, Applied Biosystems), followed by the addition of 17 µL of stock solution, to reach a total volume of 20 µL in each well. Each group consisted of three different wells, resulting in three repetitions for each group. All gene expression values were normalized to the untreated cells control group, which was considered as 100% gene expression and normalized to an unaffected housekeeping gene HPRT.

#### 2.8.3. PIP3: PI3K Protein Activation by Q-Starch/PIP3 Complexes Assessed by Western Blot 

HaCAT cells were seeded at an initial concentration of 5∙10^5^ cells/mL in a 60 mm culture dish at 37 °C and 5% CO_2_ with MEM media for 48 h to reach a confluence of ~80%. Four different groups were examined: 1. a control group of untreated cells, incubated for an additional 24 h; 2. cells treated with ten µM of PI3K inhibitor (BYL719) for 24 h of incubation; 3. cells treated with 500 nM of free PIP3 for 3 h after 24 h of incubation with 10 µM of PI3K inhibitor (BYL719); 4. cells treated with 500 nM of Q-starch/PIP3 complexes for 3 h post 24 h incubation with 10 µm of PI3K inhibitor (BYL719).

After treatment, the cell culture dish was placed on ice, and the cells were washed twice with ice-cold PBS. Lysis buffer was added to cells after the aspiration of the PBS. Adherent cells were scraped off the dish using a plastic cell scraper and transferred to microcentrifuge tubes. Then, the cells were centrifuged at 4 °C for 10 min at 14,000 rpm. The tubes were gently removed from the centrifuge and placed on ice; the supernatant was then removed and placed in a fresh tube kept on ice. Using the Bradford protein assay, the total protein concentration was measured. Amounts of 20 µL of each sample were placed in 96-well plates, and 180 µL of diluted (1:5 dilutions in DDW) Bradford solution (Bio-Rad Protein Assay Dye Reagent Concentrate) was added to each well. The plate was inserted into the Tecan Infinite M200 plate reader and analyzed at 595 nm. The samples were then diluted according to the resulting concentration and heated to 95 °C for 5 min. Equal amounts of protein, 10–20 µL (total 20 µg), were loaded into the wells of the SDS-PAGE gel, along with a molecular-weight marker. The gel was then run for 1–2 h at 100 V. Then, the gel was placed between two transfer membranes (Trans-Blot Turbo Midi 0.2 µm Nitrocellulose Transfer Packs, Bio-Rad), and air bubbles were gently pulled out. The cassette was inserted into the transfer blot machine, and the transfer was carried according to the manufacturer’s instructions. The membrane was then placed in a tray with a blocking solution consisting of 10 mL of TBST (900 mL DDW, 100 mL of tris-buffered saline 10x mixed with 1 mL tween 20), and 0.5 g of BSA. Membranes were shaken gently with the blocking solution for 1 h at room temperature. The primary antibodies of actin, P-AKT 473, and total AKT (Cell Signaling Technology, Danvers, MA, USA) were added to the membranes at a concentration of 1:1000; the tray was placed in a cold room and tilted overnight. Membranes were then washed three times, for 5–7 min on each wash, with ~5 mL of TBST. Secondary anti-rabbit antibody conjugated to horseradish peroxidase (GE Healthcare, Chicago, IL, USA) was added to 5 mL of blocking solution at a concentration of 1:20,000. Then, the secondary antibody solution was added to the membrane for 1 h of incubation at room temperature. After incubation, the membranes were washed three times with TBST for 5–7 min on each occasion. The membranes were dipped in an enhanced chemiluminescence solution (Cyanogen) and placed inside transparent nylon. The membranes were then inserted into the c300 Azure camera system (Dublin, CA, USA), and images were captured.

## 3. Results and Discussion

### 3.1. Q-Starch Synthesis and Chemical Characterization

**Figure 1:**
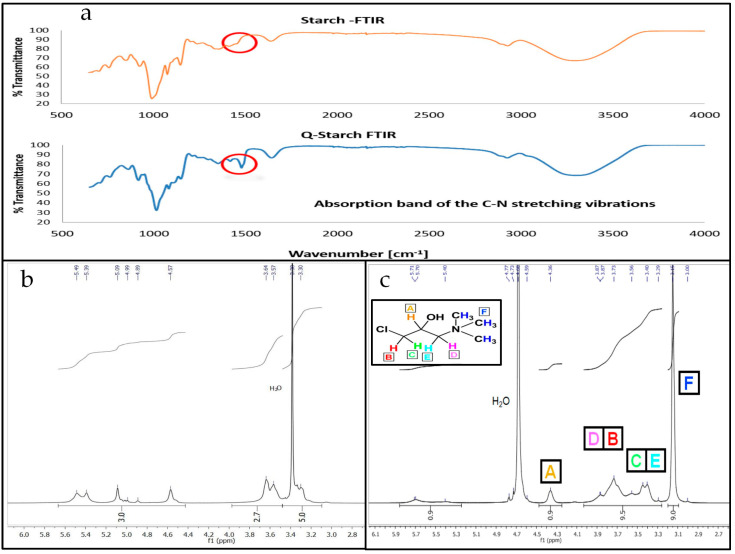
FTIR spectra of (**a**) native potato starch (above) and Q-starch (below), with the red-circle peak at 1480 cm^−1^ characterizing the CH3-N bond, confirming a successful quaternized amine agent binding to the starch backbone; (**b**) ^1^H NMR of native soluble potato starch; (**c**) CHMAC quaternization reagent with marked hydrogens (A–F). ^1^H NMR of quaternized starch (Q-starch) with the detection of the marked hydrogens.

The positively charged carrier was obtained by substituting a quaternary positively charged amine group on the starch’s backbone using a basic pH environment, as described by Amar et al. [56]. The substitution of the quaternary amine group was confirmed by an FTIR spectrum comparison between native starch and Q-starch, which revealed a peak at approximately 1480, which is a typical indication of carbon–nitrogen vibrations (Figure 1a). The binding of the reagent onto the starch was substantiated by the presence of hydrogen atoms. The NMR spectra (Figure 1b,c) revealed that all the hydrogen atoms associated with the CHMAC quaternization reagent (Figure 1c, black box) were bonded with the starch compound, native starch, as shown in Figure 1b. In addition, Q-starch’s nitrogen content was confirmed by the elemental analysis method to be 3.27% on average. 

### 3.2. Q-Starch/Cargo Complex Characterization 

The size, morphology, and surface charge properties of Q-starch/cargo complexes are all important parameters for achieving an efficient cargo delivery system. Because of this, and in line with previous studies [54,55,56], these parameters were measured at Q-starch/cargo complexes’ N/P ratios of 1–3. The concentrations of the cargo were modified according to the features and limitations of the device with which the characterization was carried out. 

#### 3.2.1. Size

**Figure 2:**
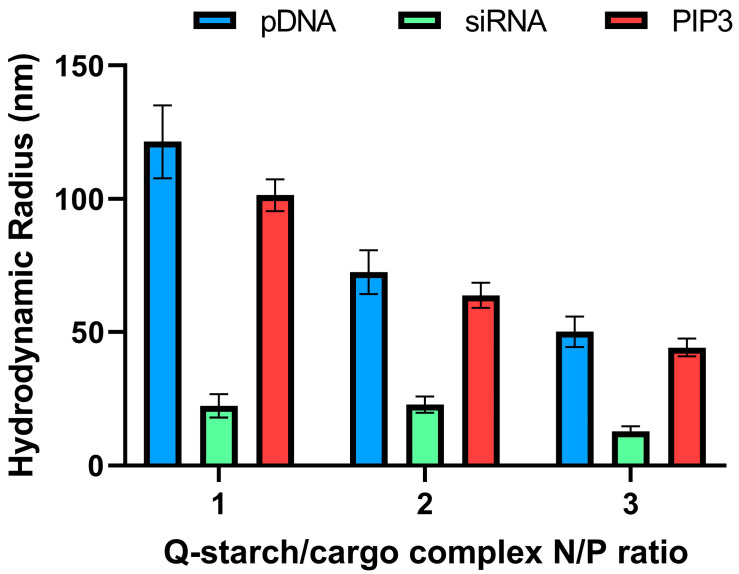
DLS measurements of hydrodynamic radii of the complexes at N/P ratios of 1, 2, and 3 for different cargos (pDNA, siRNA, and PIP3). Cargo concentrations were fixed as follows: 10 μg/mL plasmid-DNA; 250 nM siRNA; and 250 nM PIP3. Q-starch concentrations varied in line with the N/P ratio.

The radii of the complexes were measured by DLS. As can be seen in Figure 2, the hydrodynamic radii of the complexes decreased with increases in the N/P ratio. The resulting complexes were of nanometric size, suitable for penetration into the cells [63,64,65]. The size distribution of the different N/P ratios was relatively narrow (Figure S1). Comparing the three cargos, it can be seen that the largest cargo, pDNA (4700 base pairs, ~2.9 MDa), presented the largest complex. The smallest cargo, PIP3 (1126 Da), presented a larger size than siRNA (21 base pairs, ~13 KDa). pDNA is a relatively large molecule; therefore, the complex is relatively large when compared to a small molecule with a similar structure, such as siRNA. Due to its small size, siRNA appears to be more efficient in neutralizing the Q-starch positive charge, resulting in better condensation. We assumed that the size of the complex with large pDNA and that of the small PIP3 were relatively similar due to the PIP3 structure; specifically, the hydrophilic inositol head, which is connected to the quaternary amine and the hydrophobic tail, causing the Q-starch/PIP3 complex to spread due to electrostatic interactions. It can also be seen that the size of each type of complex decreases with the increase in N/P ratio. This can be explained by the linkage between the cargo’s negatively charged groups and the quaternary positively charged amine group on the Q-starch backbone, which are responsible for its folding. That is, an increase in the N/P ratio leads to higher condensation and a decrease in the complex size.

#### 3.2.2. Zeta Potential 

**Figure 3:**
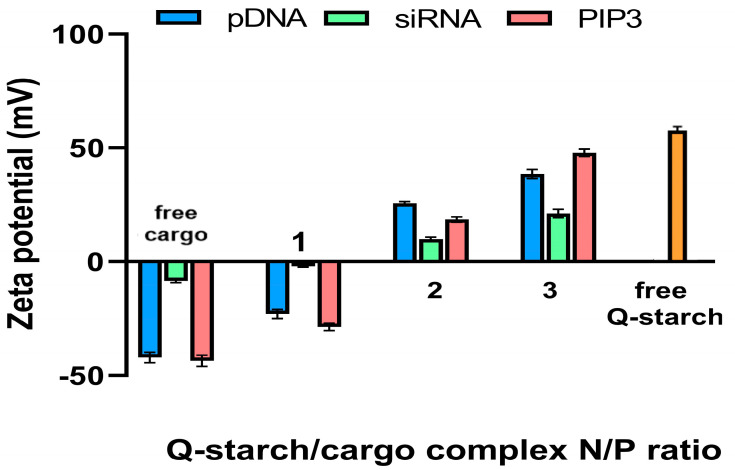
Zeta potential (mV) of the Q-starch/cargo complexes at N/P ratios of 1, 2, and 3 for the free cargos (pDNA, siRNA, and PIP3) and the free Q-starch. Cargo concentrations were fixed as follows: 5 μg/mL plasmid-DNA; 50 nM siRNA; and 50 nM PIP3 in all N/P ratios, and the Q-starch concentration was changed accordingly.

The electrical potential of the complexes was measured using a zeta sizer. One of the barriers to entry of cargo into the cells is the electrostatic repulsion between the negatively charged cargo and the negatively charged cell membrane. By attaching the cargo to the positively charged carrier at different N/P ratios, it is possible to influence the overall charge of the complex and thus overcome this barrier. As we expected, and as shown in Figure 3, the cargos pDNA, siRNA, and PIP3, in their free form, presented negative zeta potential in a range of −10 to −40 mV. Once the complexes were formed, there was an increase in the zeta potential of each of the complexes. The transition between the negative and positive potential of the complexes’ surface charge occurred between N/P = 1 and N/P = 2. The zeta potential continued to rise with increases in N/P, with the highest observed zeta potential for the free Q-starch in a range around 55 mV. 

When comparing the complexes of siRNA and pDNA, notable differences in their charge can be observed. This disparity arises from the size discrepancy between siRNA and pDNA. As siRNA is considerably smaller, it binds more extensively to the positive nitrogen units on Q-starch, resulting in a less positive charge on the siRNA complex. Conversely, the larger size of pDNA leads to steric hindrances, limiting the binding of Q-starch’s positive sites to the negative sites of pDNA. 

Similarly, PIP3, which resembles siRNA in size, possesses a structure consisting of an inositol head and a hydrophobic tail. This unique configuration may hinder the connectivity between the sites of Q-starch and PIP3. Consequently, fewer positive sites of Q-starch bind to PIP3, thereby explaining the higher positive zeta potential observed in the complex with PIP3 as compared to siRNA.

The size is affected in such a way that increasing the N/P ratio decreases the size of the complex. This can be explained by the compression of the Q-starch delivery system through stronger electrostatic bonds when increasing the N/P ratio.

#### 3.2.3. Morphology 

**Figure 4:**
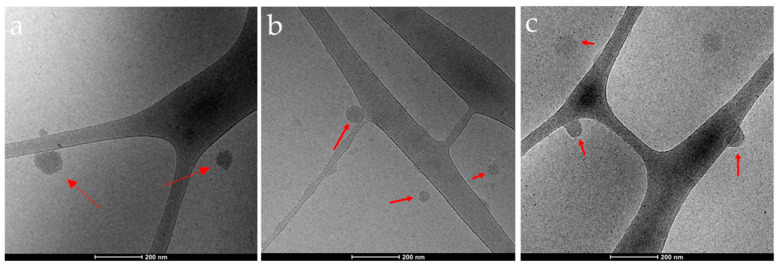
Representative cryo-TEM images of Q-starch/cargo complexes at an N/P ratio of 2 with different cargos: (**a**) pDNA at a concentration of 10 mg/mL; (**b**) siRNA at a concentration of 250 nM; (**c**) PIP3 at a concentration of 250 nM. Bar = 200 nm.

The morphology of the complexes was observed using Cryo-TEM. Figure 4 presents representative cryo-TEM images of the different Q-starch/cargo complexes at an N/P ratio of 2. Cryo-TEM images demonstrate sphere-like complexes that correspond with the DLS’s size, in a size range capable of penetrating the cell membrane. Because free cargos and free Q-starch have low electron density, these could not be detected using cryo-TEM.

### 3.3. Q-Starch/Cargo Complexes’ Cellular Localization 

To assess our hypothesis regarding cargo-complex localization in the cell, it was essential to evaluate the cellular uptake and intracellular path of the different complexes. The Q-starch/cargo complexes’ cellular uptake was examined in the HaCAT cell line, where each complex’s type was prepared and incubated with the cells, as detailed in Table 1. The concentrations of siRNA and pDNA were based on those used in previous studies [55,56], and the PIP3 concentration was chosen to be 500 nM, based on a study conducted in our laboratory (results not shown). 

**Figure 5:**
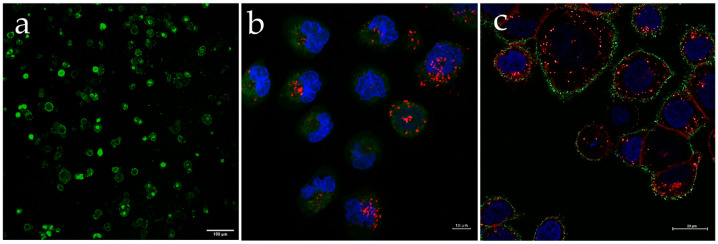
Representative live confocal images of Q-starch/cargo complexes’ cellular uptake in HaCAT cells. (**a**) HaCAT cells, 4 h after incubation with Q-starch^DTAF^/pDNA complexes, pDNA concentration at 4 mg/mL, Q-starchDTAF labeled in green, bar = 100 µm. (**b**) HaCAT cells, 4 h after incubation with Q-starch/siRNANC5Cy5 complexes, siRNANC5Cy5 concentration at 100 nM, siRNANC5^Cy5^ labeled in red, nucleus labeled in blue, and cytoplasm labeled in green, bar = 10 µm. (**c**) HaCAT cells, 4 h after incubation with Q-starch^DTAF^/PIP3 complexes, PIP3 concentration at 500 nM, nucleus labeled in blue, and cytoplasm labeled in red, bar = 20 µm.

**Figure 6:**
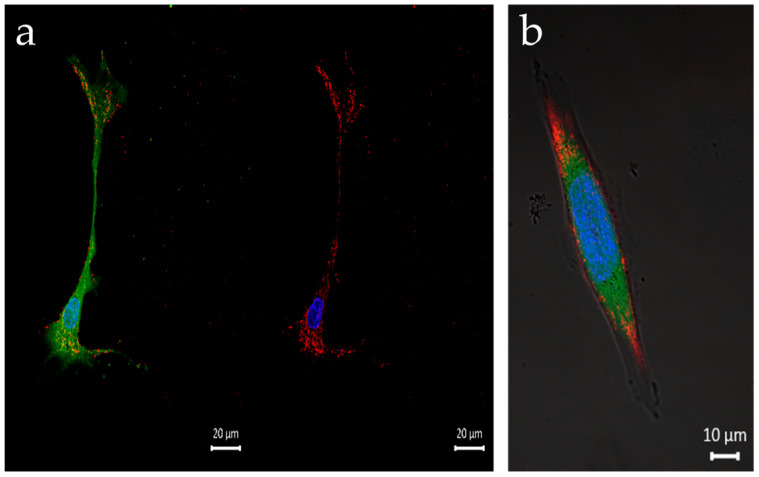
Representative live confocal images of cellular uptake 4 h post incubation with Q-starch/siRNA^Cy5^ complexes in: (**a**) melanoma cell line, right side: without cytoplasm staining, bar 20 µm; (**b**) BCC cell line, bar 10 µm. siRNA^Cy5^ labeled in red, nucleus labeled in blue, and cytoplasm labeled in green.

Figure 5 presents representative live confocal images of HaCAT cells treated with Q-starch/cargo complexes at an N/P ratio of 2. Figure 5a shows nuclear staining in green after 4 h, proving that the barrier to entry into the nucleus can be overcome by binding the pDNA to the Q-starch carrier. Further evidence supporting the entry of the complexes into the nucleus is presented in Section 3.4.1, where the biological activity of the cells is described. Specifically, the expression of GFP was observed, indicating successful transfection using Q-starch/pDNA complexes. This conclusive demonstration of cellular activity supported the idea that the complexes did indeed enter the nucleus. Figure 5b shows the Q-starch/siRNA NC5^Cy5^ complex’s cellular uptake at a concentration of 100 nM siRNA NC5^Cy5^ and an N/P ratio of 2. It can be seen (Figure 5c) that 4 h after the addition of Q-starch^DTAF^/PIP3, the complexes were dispersed on the cell membrane without entering the cytoplasm. The cellular uptake of the Q-starch/PIP3 complexes is at the membrane, where endogenous PIP3 is naturally found and active. In addition to HaCAT cells, the cellular uptake of Q-starch/siRNA complexes was observed in BCC and melanoma cells (Figure 6), in which the biological activity of Q-starch/siRNA was examined under the same conditions. In these cells, similar results were observed for the penetration of complexes into the cytoplasm, with no entry of complexes into the nucleus.

All complexes showed localization where they were naturally active. The largest particles, Q-starch/pDNA, entered the nucleus, while the smallest, Q-starch/siRNA, entered the cytoplasm and remained there without entering the nucleus for at least 4 h. The medium-sized complexes of about 150 nm diameter of Q-starch/PIP3 (i.e., similar in size to the Q-starch/pDNA complexes) remained on the cell membrane. This result indicated that the cellular uptake of the complexes is not size-correlated and is probably affected by other properties of the complex.

All the complexes in the cellular uptake experiments exhibited a positive zeta potential: 25.7 mV for Q-starch/pDNA; 9.95 mV for Q-starch/siRNA; and 18.5 mV for Q-starch/PIP3. The least-positive complex presented cellular uptake to the cytoplasm, while mid-range (Q-starch/PIP3) and high-range positivity (Q-starch/pDNA) was observed at the membrane and the nucleus, respectively. Thus, the preference of the complexes for entering different organelles in the cell cannot be explained by the difference in zeta potential.

From this experiment, it can be assumed that the localization of the complex is cargo-dependent. That is, the entry of Q-starch/pDNA into the nucleus probably results from exposed pDNA at the surface of the complexes, which allows it to bind to DNA-binding proteins, chaperons, and nuclear-importing proteins, which allow movement toward the nucleus and further penetration into the nucleus through nuclear pores [66]. siRNAs are processed by the cytoplasmic ribonuclease Dicer, located in the cytoplasm, which then activates the RNA-induced silencing complex, which is also located in the cell. We assume that enzymes and proteins in the cytoplasm are bound to the siRNA in the Q-starch/siRNA complex, keeping it in the cell’s cytoplasm [67,68,69,70]. Our assumption regarding the Q-starch/PIP3 complex’s cellular uptake at the membrane is that the complexes are “trapped” at the membrane due to the PIP3 hydrophobic tail. Despite the total positive charge of all the complexes, it is possible that the active parts of the cargo are exposed to such an extent that the proteins and cell enzymes manage to locate them and bind to them, thus leading them to the target site and keeping them there. 

In addition to the size and charge of the complexes, the spatial structure of the complexes is not a the main affector for the targeted cellular uptake since all the complexes have a globular morphological structure. 

### 3.4. Biological Activity

To determine if Q-starch might be used as a carrier for various therapeutic agents having different subcellular targets, the biological activity of the cargo was evaluated. We examined the biological activity in HaCAT and BCC cell lines for each of the complexes using a different assay for each complex.

#### 3.4.1. pDNA—Transfection GFP Assay

**Figure 7:**
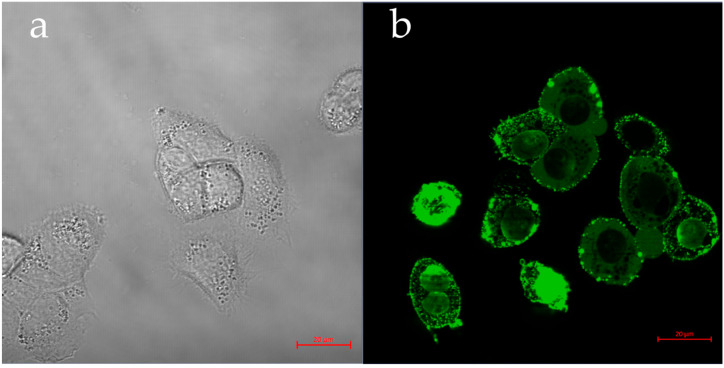
Confocal representative images of HaCAT cells: (**a**) control group after 4 h of incubation with free pEGFP-C2 pDNA at a concentration of 4 mg/mL, not exhibiting GFP (bright field); (**b**) exhibiting GFP after 4 h of incubation with Q-starch/pEGFP-C2 complexes, pDNA concentration at 4 mg/mL, bar = 20 µm.

Plasmid transfection analysis was performed to determine the efficiency of Q-starch/pDNA complexes. The successful transfection of the plasmid would demonstrate the feasibility of using Q-starch as a suitable carrier for plasmids or other therapeutic molecules whose activities occur in the cell nucleus. Figure 7 presents a live confocal image of HaCAT cells 4 h after treatment with Q-starch/pDNA complexes and a control group treated with free pDNA under the same conditions as the complex. The pDNA used was pEGFP-C2, which encodes for green fluorescence protein through transfection in the cell nucleus, leading to GFP cell staining. The transfection with the GFP pDNA was examined in an N/P ratio of 5, in line with a previous work that demonstrated higher transfection at N/P 5 than at N/P 2 [55]. At N/P 5, the complexes presented a hydrodynamic radius of 40 nm and a zeta potential of 52 mV. 

Examined groups of free starch and free pDNA did not show emission at the suitable wavelengths of GFP. Because no expression of GFP was observed in the results for free pDNA shown in Figure 7a, the cells were photographed in bright field. Evidence of transfection and entry into the nucleus is visually evident in Figure 7b, where the expression of GFP can be observed. The successful expression of the GFP gene confirmed the entry of the complexes into the nucleus, thereby demonstrating their ability to deliver the gene payload to its intended intracellular destination.

These results are proof of concept for further research of DNA-type cargo for therapeutic purposes in which Q-starch can be used as a suitable carrier. 

#### 3.4.2. siRNA—Gene Silencing

**Figure 8:**
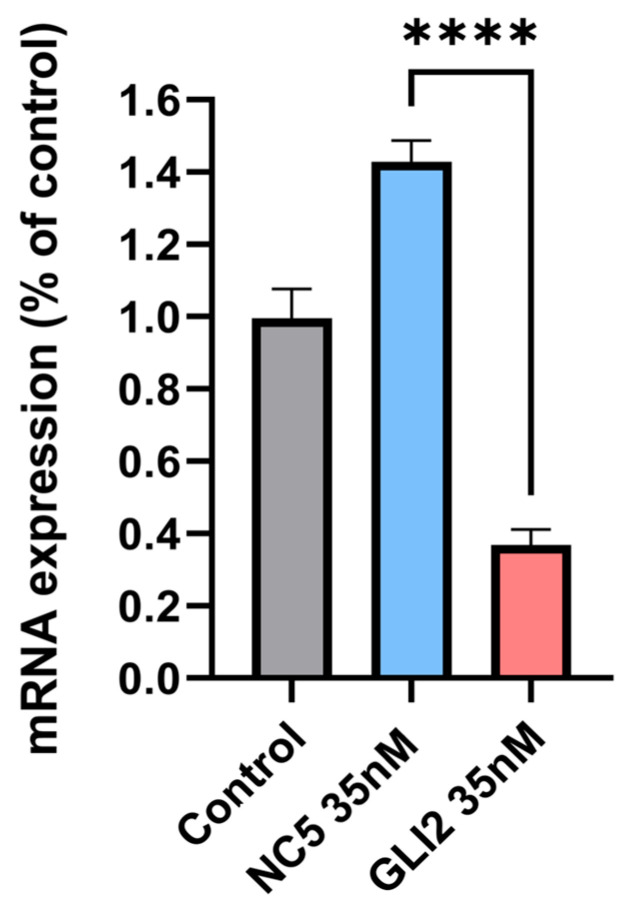
RT-PCR results of GLI2 gene mRNA expression in BCC cells, 72 h after transfection with the complexes. Three examined groups: untreated cells as a control, cells treated with Q-starch/siRNA NC5 35 nM and cells treated with Q-starch/siRNA GLI2 complexes at 35 nM. Average + SEM (n = 3).

Gene expression analysis was performed to determine the efficiency of Q-starch/siRNA complexes as gene silencing agents. Decreasing the mRNA expression of the targeted genes of interest could shed light on the potential of the complexes as a therapeutic platform. To this end, transfection experiments were performed on the BCC cell line (TE-354.T) overexpressing the GLI2 gene, based on previous high-throughput screening assessing tumor-promoting pathways in our laboratory (results not shown) and on other previous studies [71,72,73,74,75,76]. Figure 8 presents qPCR results for BCC transfected with Q-starch/siRNA^GLI2^ complexes and Q-starch/siRNA NC5 complexes, both at an N/P ratio of 2 at a concentration of 35 nM, 72 h after incubation with the complexes. It can be observed that 72 h after transfection, Q-starch/siRNA^GLI2^ complexes successfully decreased mRNA levels to 37%, resulting in efficient silencing of the over-expressed GLI2 gene, i.e., 63% gene silencing compared to untreated cells, suggesting a significant influence of our delivery system and siRNA activity on the RNA mechanism. 

#### 3.4.3. PIP3: Western Blot

**Figure 9:**
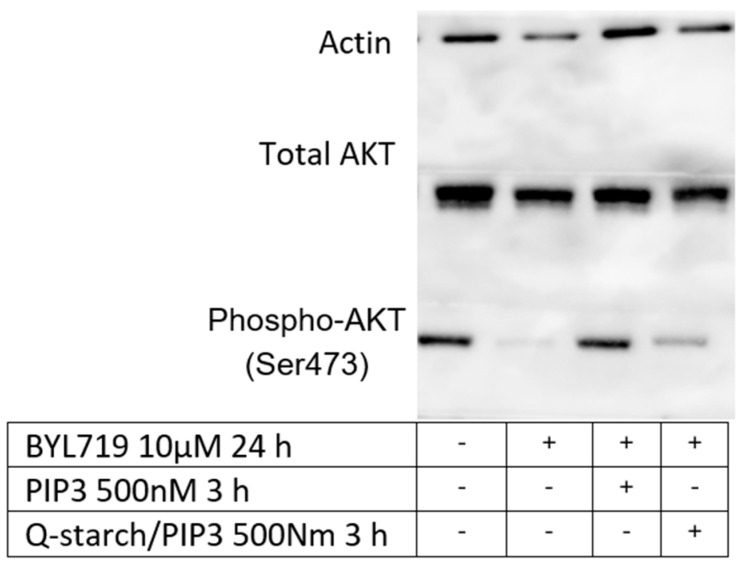
Western blot analysis of total protein lysates from HaCAT cells treated with BYL, PIP3, or Q-starch/PIP3 complexes. The panel shows detection of Actin for loading control and total AKT and phospho-AKT(Ser473) expression for assessing the effect of free PIP3 and Q-starch/PIP3 on AKT after PI3K inhibition by BYL719.

Western blot was performed to assess the effect of Q-starch/PIP3 on the phosphorylation of protein kinase B, also known as AKT. The activity of AKT was inhibited using PI3K inhibitors such as BYL719. After inhibition with BYL719, Q-starch/PIP3 or free PIP3 was added to assess whether exogenous PIP3 activated the inhibited AKT. The examination was on the phosphorylation site P-473, which was well studied and related to the activity of the AKT [77,78,79]. A greater phospho-AKT Ser473 (P-473) signal in the Western blot panel indicated higher AKT activity and vice versa. Figure 9 shows the results of the AKT activity in HaCAT cells after PI3K inhibition followed by PIP3 or Q-starch/PIP3 treatment assessed by the Western blot. The activation of P-473 can be seen clearly both in free PIP3 and Q-starch/PIP3 complexes. 

These results confirmed that the Q-starch/PIP3 complexes are located at the membrane and maintain the biological activity of PIP3. The external PIP3 activates the AKT pathway and thus can enhance migration, proliferation, and cell survival. These results demonstrate the suitability of Q-starch as a drug delivery system for therapeutic agents whose active site is the cellular membrane.

## 4. Conclusions

In this study, we present an innovative approach utilizing a highly versatile Q-starch-based carrier capable of accommodating diverse cargo types, distinguishing it from other systems that are typically tailored to a specific cargo. Our findings demonstrate the adaptability of the Q-starch carrier, which enables targeted delivery to distinct cellular sites based on the unique activity mechanisms of different cargos. Notably, Q-starch/pDNA complexes specifically localize within the cell nucleus, Q-starch/siRNA complexes reside in the cytoplasm, and Q-starch/PIP3 complexes are situated at the cell membrane. Interestingly, each cargo exhibits its biological activity at its designated target site. Additionally, our study reveals that the cellular uptake of these complexes is not influenced by their size, morphology, or zeta potential, emphasizing the decisive role of the cargo itself in dictating the final localization within the cell.

The significance and novelty of our research becomes evident as we shed light on the versatility of the Q-starch-based carrier. Our findings hold substantial implications, unveiling new possibilities for precise and efficient cargo delivery to desired cellular locations. This study paves the way for future investigations and potential breakthroughs in the targeted delivery systems of therapeutic molecules for different applications in various fields.

## Figures and Tables

**Table 1:** Q-starch/cargo complexes with examined cargo concentrations, labeling, and incubation times with the HaCAT cell line.

	Q-Starch^DTAF^/pDNA	Q-Starch/siRNA NC5^Cy5^	Q-Starch^DTAF^/PIP3
N/P ratio	2	2	2
Cargo concentration	4 mg/mL (1 nM)	100 nM	500 nM
Incubation time	4 h	4 h	4 h
Labeling	Q-starch^DTAF^/pDNA: green	Cell’s nucleus: blue; NucBlue Cell’s cytoplasm: green; Viafluor488 Q-starch/siRNA NC5^Cy5^: red	Cell’s nucleus: blue; NucBlue Cell’s cytoplasm: red; CellBrite Q-starch^DTAF^/PIP3: green

## Data Availability

Data available on request.

## Supplementary Materials

The following are available online at https://www.mdpi.com/article/10.3390/nano13131988/s1, Figure S1: Q-starch/siRNA complexes’ hydrodynamic radius distribution by DLS, at increasing N/P molar ratios 0.5, 1, 2 and 3. siRNA concentration of 250 nM.
