# Supplementary material for: Cargo-Dependent Targeted Cellular Uptake Using Quaternized Starch as a Carrier
Source: Nanomaterials (Basel). 2023 Jun 30;13(13):1988. doi: 10.3390/nano13131988 (PMC10343776; doi:10.3390/nano13131988)
Supplement: Supplementary file 1 [file nanomaterials-13-01988-s001.zip › nanomaterials-2425445-supplementary.pdf]

Cargo-Dependent Targeted Cellular Uptake Using Quaternized Starch as a Carrier

Yossi Blitsman, Chen Benafsha, Nir Yarza, Jonathan Zorea, Riki Goldbart, Tamar Traitel, Moshe Elkabets and Joseph Kost*

Supplementary

Dynamic light scattering

Size distribution of Q-starch/siRNA complexes the different N/P ratios are relatively narrow, figure S1. Hence, Complete binding of the cargos and Q-starch can be assumed.

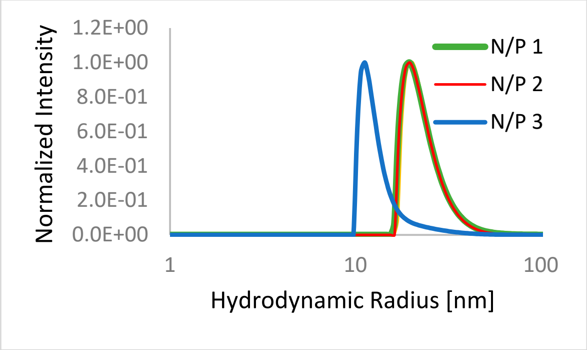

Figure S1: Q-starch/siRNA complexes' hydrodynamic radius distribution by DLS, at increasing N/P molar ratios 0.5, 1, 2 and 3. siRNA concentration of 250 nM.
